# Approaches to Improve the Oral Bioavailability and Effects of Novel Anticancer Drugs Berberine and Betulinic Acid

**DOI:** 10.1371/journal.pone.0089919

**Published:** 2014-03-10

**Authors:** Chandraiah Godugu, Apurva R. Patel, Ravi Doddapaneni, Jaganmohan Somagoni, Mandip Singh

**Affiliations:** College of Pharmacy and Pharmaceutical Sciences, Florida A&M University, Tallahassee, Florida, United States of America; UCSF/VA Medical Center, United States of America

## Abstract

**Background:**

The poor bioavailability of Berberine (BBR) and Betulinic acid (BA) limits the development of these promising anticancer agents for clinical use. In the current study, BBR and BA in spray dried (SD) mucoadhesive microparticle formulations were prepared.

**Methods:**

A patented dual channel spray gun technology established in our laboratory was used for both formulations. Gastrointestinal (GI) permeability studies were carried out using Caco-2 cell monolayer grown in in-vitro system. The oral bioavailability and pharmacokinetic profile of SD formulations were studied in Sprague Dawley rats. A549 orthotopic and H1650 metastatic NSCLC models were utilized for the anticancer evaluations.

**Results:**

Pharmacokinetic studies demonstrated that BBR and BA SD formulations resulted in 3.46 and 3.90 fold respectively, significant increase in plasma C_max_ concentrations. AUC levels were increased by 6.98 and 7.41 fold in BBR and BA SD formulations, respectively. Compared to untreated controls groups, 49.8 & 53.4% decrease in the tumor volumes was observed in SD formulation groups of BBR and BA, respectively. Molecular studies done on excised tumor (A549) tissue suggested that BBR in SD form resulted in a significant decrease in the survivin, Bcl-2, cyclin D1, MMP-9, HIF-1α, VEGF and CD31 expressions. Cleaved caspase 3, p53 and TUNEL expressions were increased in SD formulations. The RT-PCR analysis on H1650 tumor tissue suggested that p38, Phospho-JNK, Bax, BAD, cleaved caspase 3&8 mRNA expressions were significantly increased in BA SD formulations. Chronic administration of BBR and BA SD formulations did not show any toxicity.

**Conclusions:**

Due to significant increase in oral bioavailability and superior anticancer effects, our results suggest that spray drying is a superior alternative formulation approach for oral delivery of BBR and BA.

## Introduction

Non-small-cell lung cancer (NSCLC), which accounts for ∼85% of all cases of lung cancer, is a leading cause of cancer deaths worldwide. Since it is often diagnosed at an advanced stage with poor prognosis, there is need for the search of new treatment options for NSCLCs. New therapies using novel mechanisms to induce tumor cell death are needed with plants playing a crucial role as a source for potential anticancer compounds [1]. World Health organization estimated that approximately 80% of world's inhabitants rely mainly on traditional medicines for their primary health care [2]. Berberine (5,6-dihydro-9,10-dimethoxy-benzo[g]-1,3-benzodioxolo[5,6-a]quinolizinium, chloride) an isoquinoline alkaloid isolated from the rhizome, roots and stem bark of a number of Chinese herbs of Berberis species. Betulinic acid (3β, hydroxy-lup-20(29)-en-28-oic acid) is a naturally occurring pentacyclic triterpenoid of plant origin that exhibits potent antitumor, anti-retroviral, anti-malarial, and anti-inflammatory properties. Berberine (BBR) is one of the frequently used herbal medications in eastern asia and has been found to have various pharmacological activities such as antibacterial [3], antitumor [4] antioxidant, cholesterol-lowering, and anti-inflammatory [5], [6], [7], [8], [9], [10]. BBR has been shown to exhibit anticancer effects in variety of cancer cells including glioblastoma, hepatoma, melanoma, colon, breast, prostate, oral, colorectal, lung, leukemia, and osteosarcoma [11]. Further, BBR increases radiosensitivity of cancer cells, which was associated with the inhibition of HIF-1α and VEGF expression [11]. Although Betulinic acid (BA) is known to induce apoptosis and antiangiogenic response in tumor cells, the underlying mechanisms of its action remain unclear. BA inhibits growth of several cancer cell lines and tumors and the effects of BA have been attributed to its mitochondrial toxicity and inhibition of multiple pro-oncogenic factors. BA also down regulates the expression of STAT3-regulated gene products such as Bcl-xL, Bcl-2, cyclin D1 and survivin, which correlates with an increase in apoptosis as indicated by an increase in the sub-G1 cell population and an increase in caspase-3-induced PARP cleavage [12]. The antitumor effects of BA are related to the downregulation of cycli D and Bcl-xL expressions [13]. Studies have demonstrated that BA shows antimetastatic effects by inhibiting epithelial mesenchymal transition (EMT) [14]. Several studies have also suggested that BA has anti-angiogenic activity by disturbing the binding of HIF-1α and STAT3 to the VEGF promoter in hypoxic PC-3 cells [15]. BA was able to improve the effect of tumor radiotherapy under hypoxic condition [16]. Currently BA is at drug developmental stage with assistance from the Rapid Access to Intervention Development program of the NCI. Despite the demonstrated anticancer effects of BA by several mechanisms, the low aqueous solubility and poor permeability hindered the further development of this promising novel and safe anticancer agent [17]. However, clinical use of BBR and BA is limited by its poor bioavailability [18]. Similar to some other herbal products, BBR exhibits extremely low but variable plasma concentrations after oral administration in humans [19], [20]. BBR bioavailability is usually less than 1% [21], [22], the rapid biotransformation also accounts for the low plasma concentrations [10], [23]. The C_max_ of BBR in human plasma was found to be 0.4 ng/ml after a single oral dose of 400 mg [20]. In another study, C_max_ of BBR was 4 ng/ml after oral administration of 100 mg/kg in rats [24]. A high dose (up to 1.5 g/day in clinical conditions) of BBR is required which usually may trigger adverse gastrointestinal effects [10], [25], [26]. BA is orally active but due to poor bioavailability, large doses are required. It has been hypothesized that BBR co-administered with absorption enhancers might increase its bioavailability, enhance its pharmacological actions, and minimize its adverse effects [27]. Previous studies have reported various formulation approaches for BA, such as complexation with β-cyclodextrin, nanocarriers and polymeric nanoparticles. However, no studies have demonstrated the increased oral bioavailability of BA [17], [28], [29] and systematic development as well as PK/PD evaluation of BBR containing oral formulations have not been demonstrated yet.

One of the causes for low bioavailability of BBR is its poor intestinal permeation through the paracellular pathway. This pathway is actually restricted by the presence of tight junctions at the apical side of the enterocytes. Further, BBR due to quaternary ammonium cation exhibits poor water solubility, which also contributes to poor bioavailability. On the other hand BA due to extreme low aqueous solubility suffers from poor oral bioavailability. The poor aqueous solubility results in a low effective concentration and limited absorption in the gastrointestinal tract, which seriously limits its application and development as a pharmaceutical preparation. In this study, we have developed the mucoadhesive polymer coated spray dried BBR and BA formulations by using our novel dual channel spray gun which allows two streams of excipients to blend together only after spraying in micronized form. The rational for this study was to test the enhancement in bioavailability of natural compounds through our patented spray dry formulation technology using a dual channel spray gun. Hence to reinforce this technology, two model drugs which are natural products were selected due to their limited bioavailability. The poor oral bioavailability of BBR and BA was addressed by formulating the SD solid dispersion formulations containing controlled releasing polymers, solubility enhancers and permeations enhancers. The Pharmacokinetic studies were performed in rats and anticancer effects were studied in NSCLC orthotopic and metastatic lung cancer models.

## Materials and Methods

### Materials

BBR hydrochloride was purchased from Alfa Aesar (MA 01835). BA was purchased from vwr international (Suwanee, GA 30024). HPMC E3 grade was obtained from Dow Chemicals (Midland, MI, USA). Chitosan and Succinic acid were purchased from Sigma-Aldrich, Inc, USA. Hydroxypropyl Beta-cyclodextrin was obtained from Cargill Inc, (IA52406). Vitamin E TPGS NF grade was a gift sample from Antares health products, Inc (IL60174). 12 well transwell inserts were purchased from Corning, USA. Human non-small cell lung cancer cells (A549 and H1650) and Caco-2 cells were obtained from American Type Culture Collection (Rockville, MD, USA). The antibodies (cleaved caspase 3, survivin, P53, cyclin D1, MMP-9, HIF-1α and β-actin were procured from Santa Cruz Biotechnology Inc, (CA 95060). VEGF ELISA kit was purchased from Thermo Fisher scientific Inc (IL 61101). Dulbecco's modified Eagle's medium (DMEM) for Caco-2 cells, F12K medium for A549 cells and MDEM/F12K (1∶1) medium for H1650 cells were procured from Invitrogen (Grand Island, NY, USA). All culture media contained 10% fetal bovine serum, antibiotic-antimycotic solution of penicillin (5000 U/mL), streptomycin (0.1 mg/mL), and neomycin (0.2 mg/mL). Trypsin–EDTA and Hank's balanced salt solution (HBSS) and N-hydroxyethylpiperazine-N′-2-ethanesulfonate buffer solution (HEPES, pH 7.4) were obtained from Invitrogen. All other chemicals used were of analytical grade.

### In vitro anticancer effects of BBR

NSCLC cells A549 and H1650 were plated in 96 well plates at 10,000 cells/well density. After 24 h, cells were treated with various concentrations of BBR and BA. DMSO (0.1%) treated cells were considered as control cells. After 72 h treatment, cells were fixed in 0.25% gultaraldehyde and stained with 0.05% crystal violet staining. Percent of cell viability or cell kill was calculated by considering DMSO treated control cells as 100% viable (zero % cell kill).

### Preparation of SD formulation

BBR SD formulations were prepared with optimized proportions as follows: BBR 1.5 gm, HPβ-CD (Hydroxy propyl Beta-cyclodextrin) 1.5 gm, HPMC (Hydroxy propyl methyl cellulose) 1 gm, chitosan 1 gm, vitamin E TPGS 500 mg, glycerol 100 mg and menthone 100 mg. HPβ-CD and BBR were dissolved in 50 ml of water. In another container, 2% chitosan was added to HPMC suspension. The optimized formulation was spray dried by using our patented dual channel spray drying system. One of the liquid channels contained SD formulation containing homogenous mixture slurry and in another liquid channel 2% chitosan solution was used, which will coat a chitosan layer on the microparticles during the dual channel spray drying process. BA SD formulations were prepared using optimized formulation composition : BA 1 gm, HPβ-CD 1.5 gm, carbopol 0.5 gm, vitamin E TPGS 1 gm, volpo-20 250 mg and mannitol 1.5 gm. BA and vitamin E TPGS were dissolved in 20 ml ethanol. In another container HPβ-CD, mannitol, volpo-20 and carbopol were mixed in 30 ml of water. The final liquid composition slurry was served as one of the liquid feed and in another liquid feed 2% Carbopol was used as coating liquid, which will coat bioadhesive carbopol layer on the microparticles during the dual channel spray drying process. Both liquids through separate pumps will be fed at pulsatile action simultaneously at steady flow rate 3 ml/min liquid feed rate and resultant mixture was SD at inlet air temperatures at 130°C.

### Characterization of SD formulations

DSC (Differential Scanning Calorimetry) was recorded on a Perkin-Elmer DSC 7 (Perkin-Elmer, Norwalk, CT, USA). Samples of approximately 5–10 mg were weighed and hermetically sealed in aluminum pans (Kit 0219-0062, Perkin-Elmer Instruments, Shelton, CT, USA). The samples were heated from 30°C to 300°C at a ramp rate of 10°C/min and a nitrogen purge at a flow rate of 20 ml/min. An empty aluminum pan was used as the reference. The thermograms were developed by plotting temperature on the x-axis and heat flow (Endotherm up) on the y-axis. The surface morphology of SD microparticles was studied by scanning electron microscopy (SEM). Samples were placed on carbon support and analyzed by Zeiss 1540 XB field emission SEM (NY 10594).

### In vitro permeability studies

In vitro drug permeability studies were evaluated in Caco-2 cells according to the standard procedures, the detailed procedure was given as Data S1.

### In vitro drug release studies

In vitro drug release study was conducted in PBS (pH 5.8) containing 1% volpo. Briefly, 50 mg of free drug or equivalent amount of SD form of BBR and BA were dispersed in 500 ml of release medium and the drug release profile was studied at pH 5.8. Release studies were performed according to the USP type II paddle method, at 37±0.5°C while stirring constantly at 50 rpm. Samples were withdrawn at different time points, centrifuged and drug content in the samples was analyzed by spectrometric method of analysis. The withdrawn samples were replaced by equal volumes of fresh medium maintained at the same temperature. Except replacing the 1% Volpo with 2.5% SLS, similar conditions were maintained for the in vitro drug release studies of BA in free drug and SD forms. The released BA concentrations were measured by established HPLC method.

### Animals

The laboratory murine model has been used extensively for lung cancer research. Female, 6-week old, athymic Nu/nu mice were purchased from Harlan Inc. (Indianapolis, IN). Sprague Dawley rats were used for the pharmacokinetic studies of BBR and BA. The mice and rats were housed and maintained in specific pathogen-free conditions in a facility approved by the American Association for Accreditation of Laboratory Animal Care. Food and water were provided ad libitum to the animals in standard cages. Animals were maintained at standard conditions of 37°C and 60% humidity. All experiments were done in accordance with the guidelines of the Institutional Animal Care and Use Committee (IACUC) at Florida A&M University. The IACUC of Florida A&M University approved this animal study. Animals were acclimatized for 1 week prior to the tumor studies.

### Pharmacokinetic studies

The oral bioavailability and pharmacokinetic profile of SD formulations of BBR and BA were studied in Sprague Dawley rats at the dose of 100 mg/kg orally. Rats were fasted overnight before the start of the experiments and randomly divided into two experimental groups. After the drug administration, blood samples (250 µl) were withdrawn from tail veins at predetermined time points (0, 0.25, 0.5, 1, 2, 4, 6, 8, 12, 24, and 48 h. The blood samples were placed in heparinized microvet blood collection tubes and plasma was obtained by centrifugation at 10,000 rpm for 10 min and then stored at −80°C until analysis. BBR and BA were extracted from the plasma by protein precipitation method and extracted samples were dissolved in mobile phase and samples were analyzed by HPLC method. The pharmacokinetic parameters such as area under the curve (AUC), C_max_, t_1/2_, t_max_, MRT, etc were analyzed. Pharmacokinetic parameters were analyzed using non-compartmental techniques with WinNonlin® 5.0 software (Pharsight Corporation, Mountain View, CA, USA). SHAM analysis (i.e., Slope, Height, Area, and Moment) utilized plasma concentration-time data to estimate the area under the curve (AUC), apparent terminal elimination rate constant (λz), terminal elimination half-life (t_1/2_) and the area under the first moment of the plasma concentration–time curve (AUMC). The AUC was calculated for each rat using the piecewise log trapezoidal areas and extrapolated to infinity by dividing λ_z_ into the last measured plasma concentration (i.e. C_last_/λ_z_). From the values of AUC0_0-∞_ and AUMC_0-∞_, the clearance (CL), mean residence time (MRT) and volume of distribution at tissue equilibrium (V_ss_) were calculated based on noncompartmental monoexponential assumptions as: CL = Dose/AUC, MRT≅AUMC/AUC and Vss = CL×MRT. The noncompartmental parameters were calculated for each rat before averaging dose groups. The pharmacokinetic parameters such as AUC, C_max_, t_1/2_, t_max_, MRT, etc were compared between the free drug and SD formulations. The final formulation with improved oral bioavailability in PK studies was used for the anticancer evaluations.

### Orthotopic (A549 cells) and metastatic (H1650 cells) lung cancer models

A549 cells were grown in F12K Medium containing 10% FBS and standard antibiotic mix at 5% CO_2_ and 37°C. For orthotopic lung tumor model, mice were anesthetized with Isoflurane and a 5 mm skin incision was made to the left chest, 5 mm below the scapula. Hamilton syringes (1 ml) with 28-gauge hypodermic needles were used to inject the cell inoculums through the sixth intercostal space into the left lung. The needle was quickly advanced to a depth of 3 mm and quickly removed after the injection of the A549 cells (2 million/mouse) suspended in 100 µL PBS (pH 7.4) into the lung parenchyma. For metastatic lung tumor models H1650 cells (2 million in 100 µL PBS) were intravenously administered to mice via tail vein. Our previous studies reported that tail vein injection of H1650 cell resulted in the formation of metastatic tumor nodules in lung. Only cell suspensions of >95% viability as determined by tryphan blue exclusion were used. Wounds from the incisions were closed with surgical skin clips. Animals were observed for 45 to 60 min until fully recovered. These animals develop lung cancer within 14 days after the cell inoculation. Drug treatment was started 2 weeks after the tumor cell implantation. BBR free drug or SD formulation was given orally at the dose of 100 mg/kg daily for 3 weeks. In case of BA anticancer studies, free drug and BA-SD were administered to orthotopic and metastatic lung tumor models at the dose of 100 mg/kg daily for 3 weeks. Body weights were monitored throughout the study. Animal survival rates were recorded during the study. The lung weights and tumor volumes were used for assessment of therapeutic activity of the treatments. After sacrificing the animals lung weights and tumor nodules were recorded in metastatic tumor models.

### Western blot analysis

**Western blot analysis was performed to study the expression of** Bcl-2, cleaved caspase 3, survivin, cyclin D1, p53, MMP-9, HIF-1α. The detailed procedure was given as Data S1.

### IHC for cleaved caspase 3 and CD31

Formalin fixed tumors were processed for automatic histopathological procedures. The detailed procedure was provided in Data S1 [30].

### TUNEL assay

Terminal deoxynucleotidyl transferase-mediated dUTP nick end labeling (TUNEL) assay was used to study the DNA fragmentation or apoptosis detection. TUNEL assay was performed on 4–5 µm thick sections as per the kit manual. Formalin-fixed tumor tissues were embedded in paraffin and sectioned (4–5 mm thick). DeadEndTM Colorimetric Apoptosis Detection System (Promega, Madison, WI) allows the recognition of apoptotic nuclei in paraffin-embedded tissue sections fixed on slides according to the manufacturer's protocol. Briefly, the equilibration buffer was added to slides and incubated for 10 minutes followed by 10-minute incubation in 20 mg/ml proteinase K solution. The sections were washed in PBS and incubated with TdT enzyme at 37°C for 1 h in a humidified chamber for incorporation of biotinylated nucleotides at the 3-OH ends of DNA. The slides were incubated in horseradish peroxidase-labeled streptavidin to bind the biotinylated nucleotides followed by detection with stable chromagen DAB. The images on the slides were visualized with an Olympus BX40 light microscope equipped with a computer-controlled digital camera (DP71, Olympus Center Valley, PA, USA). Three slides per group were stained and apoptotic cells were identified by dark brown cytoplasmic staining.

### Histopathology of lung tumors and ileum

Orthotopic lung tumors and mice small intestine segments (ileum) were processed for normal histopathological procedures and 5–10 µm sections were made. After deparaffinization and rehydration, sections were stained with Hematoxylin and Eosin for normal histological evaluation. Similarly, small intestine segments were processed and stained with H&E and visualized under normal microscope.

### Quantification of VEGF by ELISA

VEGF levels were estimated in plasma, tumor tissue homogenates by ELISA method. Tumors were homogenized in RIPA lysis buffer (20 mM Tris-HCl pH 7.5, 150 mM NaCl, 1% Triton X-100) containing proteinase inhibitors (Complete Proteinase Inhibitor Cocktail Tablets, Roche, Indianapolis. IN). Tissue debris was pelleted and the resulting supernatant was used in subsequent analysis. Total protein concentration was determined with a BCA protein assay (Pierce, Rockford, IL). VEGF concentrations were determined according to the manufacturer's instructions. A minimum of 3 tumors per group were analyzed in duplicate [30].

### Real time PCR assay

Total RNA was extracted from tumors using RNeasy Kit and quantified by UV method using Nanodrop system. RNA was converted to complementary DNA using RT^2^ First Strand Kit and RT^2^ SYBR Green ROX qPCR Mastermix as per manufacturer's instructions [31]. p38, Phospho-JNK, Bax, Bcl-2, cleaved caspase 8, cleaved caspase 3, survivin and BAD, mRNA expressions amplification was performed on an ABI 7300 RT-PCR (Applied Biosystems, Carlsbad, CA, USA) and data analysis done with a PCR Array Data Analysis Software (SA Biosciences, Valencia, CA, USA). The mRNA levels of p38, Phospho-JNK, Bax, Bcl-2, cleaved caspase 8, cleaved caspase 3, survivin and BAD and β-actin were calculated. The β-actin was a normalization control for gene expression.

### Statistics

Data was presented as the mean ± standard error of mean (sem). Statistical differences were evaluated by Student's t-test or one way analysis of variance (ANOVA) followed by Tukey's test. The criterion for statistical significance was set at *p*<0.05.

## Results

### In vitro cytotoxicity and clonogenic assay

BBR produced concentration dependent cytotoxicity on A549 and H1650 NSCLC cells. Cell viability was significantly decreased with increased concentration of BBR (Figure S1). In both A549 and H1650 cells, 72 h exposure resulted in significant cytotoxicity compared to 24 & 48 h exposure (data now shown). The IC_50_ values of BBR on A549 and H1650 cells after 72 h exposure was found to be 6.45±0.46 and 8.39±1.03 µM, respectively. This in vitro cytotoxicity data suggest that A549 and H1650 cells are sensitive to BBR treatment. The clonogenic assay also demonstrated the effect of BBR on colony formation. The number of colonies and colony sizes were significantly decreased in 1, 3 and 10 µM BBR treated groups. Further, BBR in SD form also exhibited significant colony inhibition (Figure S2). Both invitro cytotoxicity and clonogenic assay suggest that BBR has potential anticancer activity on NSCLCs. The in vitro cytotoxicity of BA suggested the BA has anticancer cancer activity on NSCLCs. The IC_50_ values of BA were found to be 8.92±1.68 and 7.25±1.54 µg/ml, respectively on A549 and H1650 cells.

### Characterization of SD formulations

**Figure 1:**
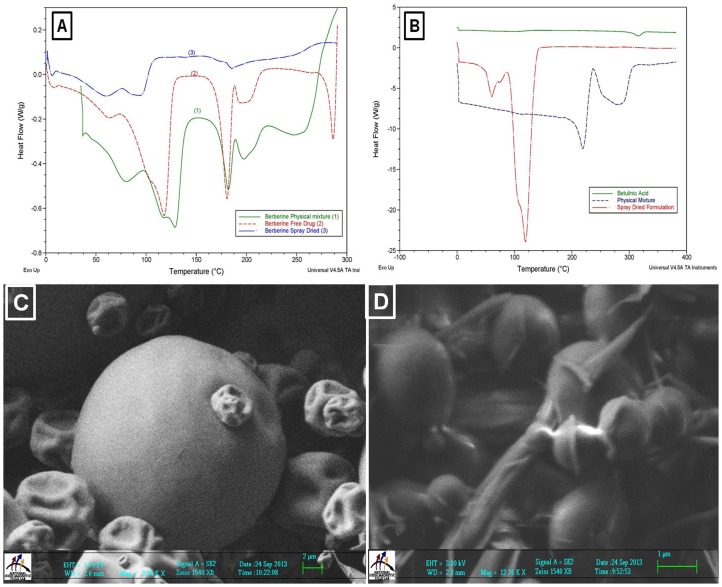
Characterization of BBR-SD and BA-SD formulations. A) DSC analysis of BBR free drug, Physical mixture and BBR-SD formulation. B) DSC analysis of BA free drug, Physical mixture and BA-SD formulation. Scanning electron microscopic analysis of C) BBR-SD and D) BA-SD formulation by using Zeiss FESEM.

In order to characterize the thermal behavior of the BBR-SD formulations, differential scanning calorimetry (DSC) was performed. It was observed that BBR exhibits a peak melting endotherm at ∼180°C. The appearance of a peak corresponding to the melting of BBR was observed in the thermogram of the physical mixture, whereas in the SD formulations it was disappeared and the thermal behavior of BBR-SD exhibited no such BBR characteristic thermal behavior at any temperature intervals. The DSC data indicates that BBR was well dispersed in SD formulation, that is why the characteristic drug's thermogram trough was disappeared (Figure 1A). Similarly the DSC analysis of BA-SD formulations was performed. BA showed characteristic peak melting endotherm at ∼325°C, where as in SD formulation, the characteristic BA endothermal peak was disappeared, suggesting the incorporation of BA in SD microparticles (Figure 1B). The scanning electron microscopic analysis indicated that upon spray drying of liquid dispersions in to solid dispersions resulted formation of microparticles with approximate sizes of 1–5 µm. The BBR-SD microparticles sizes were found to be spherical in shape with particle size distribution of 1–5 µm size (Figure 1C), while in case of BA-SD formulations, these microparticles were found to be in the size ranges of 1–3 µm (Figure 1D).

### Drug release studies

**Figure 2:**
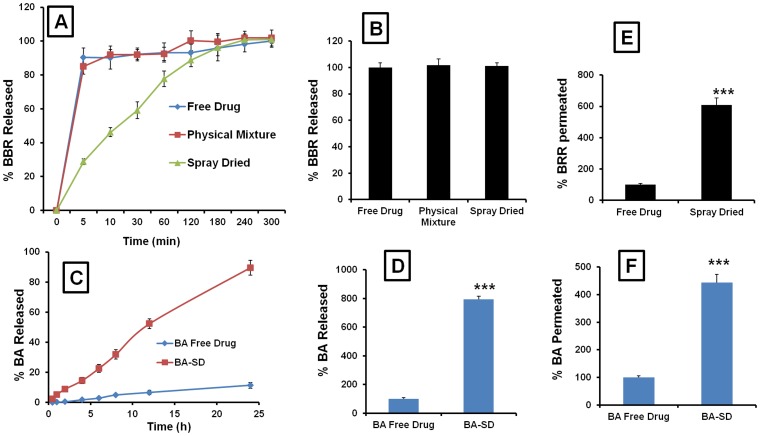
In vitro evaluation of BBR-SD and BA-SD formulations. A) In vitro drug release profile of BBR in free drug, physical mixture and SD form. B) Final drug release profile of BBR in free drug, physical mixture and BBR-SD formulation forms after 48 h. C) In vitro release profile of BA in free drug and SD form. D) % final drug dissolution profile after 24 h in BA free drug and BA-SD formulations. E) Caco-2 permeability of BBR in free drug and BBR-SD form, % of BBR permeated from apical to basolateral compartment. F) Caco-2 permeability of BA in free drug and BA-SD form, % of BA permeated from apical to basolateral compartment. Each data point was represented as mean±sem (n = 3–4). ***p<0.001 Vs respective free drug groups.

In vitro drug release studies indicated that after 5 h, the percent of BBR released from free drug, formulation excipients physical mixture and SD formulation groups was found to be similar. Approximately 100% of BBR was released in all three groups. Due to slow release from the SD microparticles, BBR in SD form followed control release pattern (Figure 2A&B). In case of BA release studies, after 24 h, free drug resulted in only 11.3% drug release, where as in BA-SD, 89.6% of drug was released, (7.92 fold increase in the drug release was observed in BA-SD compared to free drug, Figure 2D). Due to molecular dispersion of BA in SD matrix polymers, the significant increase in the BA release or dissolution was observed (Figure 2C&D).

### In vitro permeability studies

The TEER values and mean permeability values of paracellular control Lucifer Yellow were >350 Ω•cm^2^ and <0.15×10−6 cm/s respectively which was within normal limits, thus confirming paracellular integrity of monolayers. The average Peff, A–B across caco-2 cell monolayers was approximately 0.29±0.08×10−6 cm/s and 1.76±0.19×10−6 cm/s, respectively in BBR free drug and BBR-SD formulations. The Caco-2 permeability experiment revealed that compared to BBR free drug in SD formulations 6.08 fold increase in the drug permeability was observed (Figure 2E). The TEER values were decreased upon treatment with BBR-SD formulations. These values were decreased from 350 Ω•cm^2^ before SD formulation application to 220 Ω•cm^2^ (data not shown). This decrease could be due to the opening of the tight junctions by chitosan and other permeation enhancers used for the preparation of SD formulation. However, the decreased TEER values were recovered back to the original levels after washing the applied formulation from Caco-2 cell monolayers. The TEER values after multiple washes of the monolayers were found to be 345 Ω•cm^2^, indicating the reversible nature of this permeation enhancers to open the tight junctions. The BBR-SD formulation components did not affect the viability of the Caco-2 cell monolayers. The viable cells counting on following 2 h treatment of SD formulation was found to be not altered (data not shown). This suggests that increased permeation in SD formulation is due to opening of the tight paracellular junctions, but not because of toxicity to the Caco-2 monolayer. Caco-2 drug absorption studies indicated the increased permeability in SD groups. With BA-SD group significant increase in the BA permeation was observed in Caco-2 studies compared to free drug (Figure 2F). The TEER values were found to be not altered before and after treatment of BA-SD formulation to monolayers, which suggest the integrity of Caco-2 monolayers.

### Pharmacokinetic study

**Figure 3:**
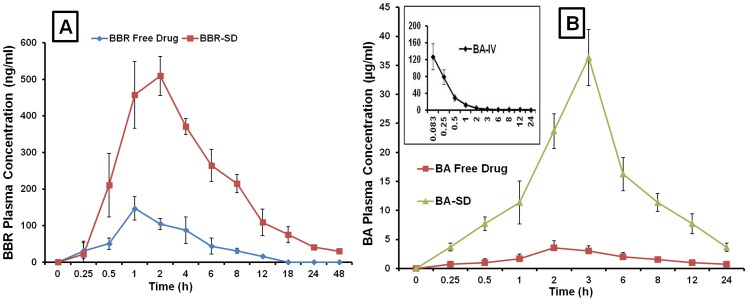
Pharmacokinetic profiles of BBR-SD and BA-SD formulations. A) Plasma concentration time profile of BBR free drug and BBR-SD formulation in Sprague Dawley rats. B) Plasma concentration time profile of BA free drug and BA-SD formulation in Sprague Dawley rats. In both the studies, rats were administered at the dose of 100 mg/kg, orally. Figure 3B also shows the plasma concentration and time profiles of BA upon intravenous administration. Each data point was represented as mean±sem (n = 3–4).

Oral administration of BBR-SD formulations (100 mg/kg) in SD rats resulted in significant increase in the oral absorption when compared to BBR free drug. The plasma C_max_ concentration in BBR-SD was found to be 509.31±39.87 ng/ml, while in free drug it was 146.87±21.76 ng/ml, 3.46 fold increase in the C_max_ levels were observed in BBR-SD compared to free drug. The area under the curve (AUC)_0-∞_ was found to be significantly higher in SD formulation when compared to free drug. The (AUC)_0-∞_ in BBR-SD group was 5724.39±453.67 ng.h/ml, while in free BBR group the values were 819.35±81.96 ng.h/ml. The 6.98 fold increase in the (AUC)_0-∞_ was observed in BBR-SD. Plasma half life (t_1/2_) was also significantly increased in spay dried group. The BBR free t_1/2_ was found to be 5.95±0.47 h, while in BBR-SD group the plasma half life was found to be 14.92±1.32 h. The mean residence time (MRT) in BBR-SD formulation was 18.33 h±3.51 h, whereas in free BBR groups it was 6.85±1.68 h, suggesting the sustained release behavior of BBR-SD formulation (Figure 3A and Table 1). The plasma C_max_ concentration in BA-SD was found to be 4.54±0.25 µg/ml, while in free drug resulted in 1.16±0.22 µg/ml. Approximately 3.90 fold increase in the C_max_ concentration was observed in BA-SD groups. The (AUC)_0-∞_ was found to be significantly higher in SD formulation when compared to free drug, 7.41 fold increase in the (AUC)_0-∞_ was observed in BA-SD compared to free drug. The (AUC)_0-∞_ in BA-SD group was 53.86±7.79 µg.h/ml, while in free BA group the values were 7.26±1.65 µg.h/ml. Plasma half life was also significantly increased in BA-SD group. The BA free drug t_1/2_ was found to be 12.33±3.68 h, while in BBR-SD group the plasma half life was found to be 15.18±4.37 h. The MRT in BA-SD formulation was 15.96±4.78 h, whereas in free BBR groups it was 15.05±3.86 h, suggesting the sustained release behavior of BA-SD formulation. The improved pharmacokinetic parameters such as increased C_max_, t_1/2_, AUC and MRT in BBR-SD and BA-SD suggest the superior oral bioavailability profile of SD BBR and BA formulations (Figure 3B and Table 1).

**Table 1:** Pharmacokinetic profile of BA-SD and BBA-SD.

Parameter	BA free drug	BA-SD		BBR free drug	BBR-SD
**Cmax (µg/ml)**	1.16±0.22	4.54±0.25	**Cmax (ng/ml)**	146.87±21.76	509.31±39.87
**t_max_ (h)**	2.36±0.38	3.17±0.85	**t_max_ (h)**	1.41±0.67	2.86±0.91
**AUC (0-∞) µg.h/ml**	7.26±1.65	53.86±7.79	**AUC (0-∞) ng.h/ml**	819.35±81.96	5724.39±453.67
**AUMC (0-∞) µg.h^2^/ml**	116.04±23.13	810.91±68.08	**AUMC (0-∞) ng.h^2^/ml**	5619.03±651.24	104959.60±987.81
**t_½_ (h)**	12.33±3.68	15.18±4.37	**t_½_ (h)**	5.95±0.47	14.92±1.32
**Vd (ml/kg)**	122.43±15.86	93.18±12.23	**Vss (ml/kg)**	1048.39±106.35	376.15±24.39
**CL (ml/kg/h)**	6.78±1.05	0.92±0.54	**CL (ml/kg/h)**	122.04±16.88	17.46±2.70
**MRT (h)**	15.05±3.86	15.96±4.78	**MRT (h)**	6.85±1.68	18.33 h±3.51

The pharmacokinetic parameters of BA free drug and BA-SD formulations groups after oral administration of 100 mg/kg.

### Anticancer activity of BBR-SD and BA-SD in A549 NSCLC orthotopic and H1650 metastatic lung cancer model

**Figure 4:**
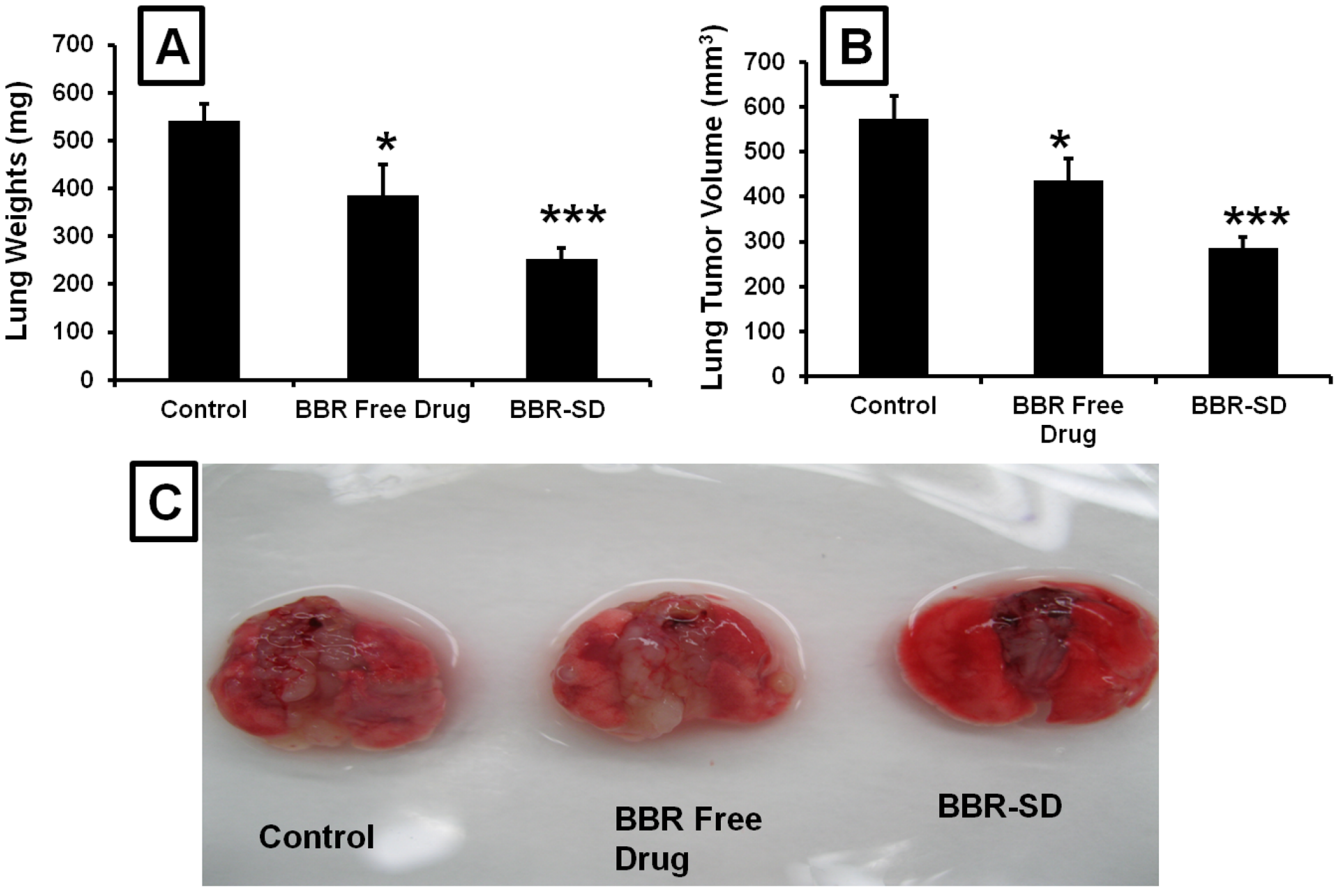
Anticancer effects of BBR-SD formulations in orthotopic lung cancer model. A) Lung tumor weights after treatment with BBR free drug and BBR-SD formulations in A549 orthotopic lung tumor models, B) Lung tumor volumes after treatment with BBR free drug and BBR-SD formulations in A549 orthotopic lung tumor models, C) Representative lung tumor images taken from control, BBR free drug and BBR-SD treated (3 weeks daily 100 mg/kg dose) animals. Each data point was represented as mean±sem (n = 6–8). *p<0.05 and ***p<0.001 Vs respective untreated control groups.

**Figure 5:**
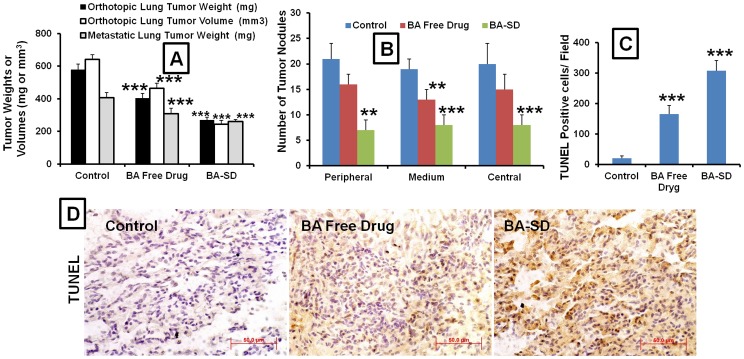
Western Blot analysis of orthotopic lung tumors lysates. A) Western blot images of Bcl-2, survivin, cyclin D1, p53, MMP-9, HIF-1α and β-actin expression in control, BBR free drug and BBR-SD formulation treated tumor lysates. B) Densitometric analysis of β-actin relative expression of C) Bcl-2 and C) survivin. Each data point was represented as mean±sem (n = 3–4). ***p<0.001 Vs respective untreated control groups.

In mice A549 orthotopic lung cancer models, treatment with BBR free drug and BBR-SD formulations resulted in significant decrease in the lung tumor weights. The tumor weights were found to be 540.60±36.18, 385.77±63.42 and 253.49±21.99 mg respectively, in untreated control, BBR free drug and BBR-SD formulation groups (Figure 4A). When compared to control tumors, BBR free drug treatment resulted in 1.40 fold reduced tumor weights, where as BBR-SD formulation resulted in 2.13 fold decrease in the lung tumor weights. Similarly, significant reductions in tumor volumes were also observed (Figure 4B). Compared to untreated control groups, 1.31 and 2.00 fold, respective decrease in the tumor volumes were recorded in BBR free drug and BBR-SD formulation treated lung tumors. Both tumor weights and volumes suggested that compared to BBR free drug, BBR-SD formulations resulted in 1.52 fold increased anticancer effects (Figure 4A&B). The observed data suggest that BBR in free drug and BBR-SD form exerts anticancer effects in NSCLC models and BBR-SD has superior anticancer effects. The representative orthotopic lung tumors were shown in Figure 4C. Therefore, the increased oral pharmacokinetics profile observed in BBR-SD formulations translated into the increased pharmacodynamic anticancer effects. The lung tumor weights were significantly reduced in BA free drug and BA-SD formulations. The average lung tumor weight in control group was found to be 578.51±41.69 mg, these weights were decreased to 404.31±29.50 and 269.39±23.67 mg respectively, in BA free drug and BA-SD treated groups. Approximately 31.11 and 53.40% reduction in the tumor weights was observed in BA free drug and BA-SD groups compared to untreated control tumors (Figure 5A). The tumor volumes were also significantly decreased in BA free drug and BA-SD treated groups. Compared to control tumor volumes, in BA free drug treated groups the tumor volumes reduced by 27.72%, whereas in BA-SD treated animals, tumor volumes reduced by 62.05% (Figure 5A). Similarly, in metastatic tumor models also tumor weights in BA free drug and BA-SD formulation treatments found to be reduced significantly to 75.94 and 63.91% respectively, compared to untreated control groups (Figure 5A). In both orthotopic and metastatic lung tumor models, BA-SD produced superior anticancer effects compared to BA free drug groups, in terms of lung tumor weights and tumor volumes show 1.49 and 1.90 fold superior in BA-SD treated groups. In metastatic lung tumor models, the numbers of tumor nodules in the peripheral, medial and central lobes were significantly decreased in BA free drug and BA-SD formulation treated groups. In the peripheral lobes, the tumor nodules were decreased by 1.31 and 3.00 fold in BA free drug and BA-SD treated animals. Further, BA-SD treatment resulted in decrease in tumor nodules (2.28 fold superior) compared to BA free drug treated groups (Figure 5B). Similarly, in medial and central lobes also, BA-SD formulation treatment resulted in more decrease in lung tumor nodules compared to BA free drug treated animals. In medial and central lobes, BA-SD produced 1.62 and 1.87 fold superior reduction in tumor nodules compared to BA free drug treated groups (Figure 5B). All these results suggest that BA-SD exhibit super anticancer activity in orthotopic and metastatic lung tumor models. The increased oral bioavailability observed in BA-SD formulations might be the possible explanation for the increased activity.

### Western blot

**Figure 6:**
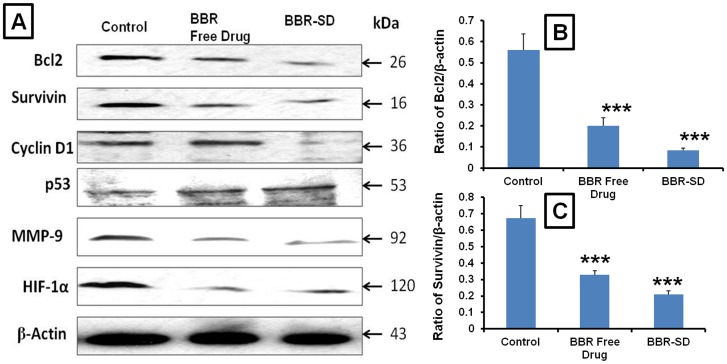
Western blot Densitometric analysis. A) Densitometric analysis of β-actin relative expression of A) cyclin D1, B) p53, C) MMP-9 and D) HIF-1α. Each data point was represented as mean±sem (n = 3–4). ***p<0.001 Vs respective untreated control groups.

**Figure 7:**
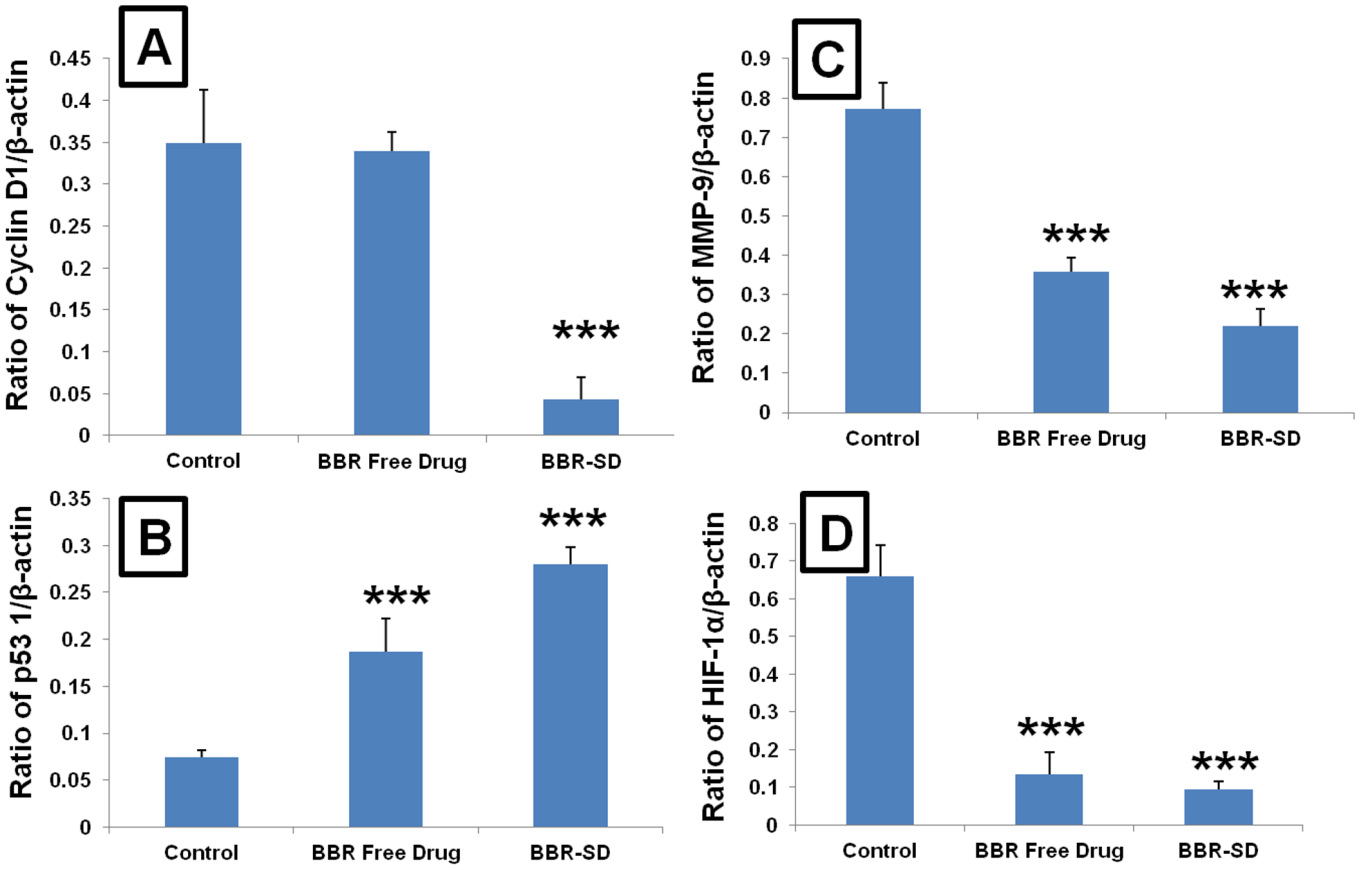
Immunohistochemical (IHC) analysis of orthotopic lung tumor sections. IHC analysis of tumor sections collected from untreated control, BBR free drug and BBR-SD formulation treated animals. First row of images shows the cleaved caspase-3 expression in different groups. The brown color stained cells indicate the cleaved caspase-3 specific positive cells. Second row shows the CD31 expression, the brown colored cells suggest the MVD positive cells. Third row shows the TUNEL assay, brown color stained cells indicate the apoptotic positive cells.

Further, the mechanistic studies also confirmed the superior anticancer cancer effects of BBR-SD formulation compared to BBR free drug treated groups. Western blot analysis of the antiapoptotic marker Bcl-2 expressions suggested that in SD groups Bcl-2 was significantly down regulated in BBR-SD group compared to untreated control and BBR free drug treated groups (Figure 6A&B). The densitometric analysis of western blot bands revealed that beta actin relative Bcl-2 expressions were found to be reduced 2.79 and 6.64 fold, respectively in BBR free drug and BBR-SD groups compared to untreated control tumors. The BBR-SD produced 2.37 fold higher repression in the Bcl-2 expression compared to BBR free drug, suggesting the superior anticancer effects of BBR-SD in lung cancer. Similarly, the cell survival marker survivin expression was also significantly down regulated (2.03 and 3.19 fold) in BBR free drug and BBR-SD groups compared to control groups (Figure 6A&C). The cell proliferating related cyclin D1 expressions were found to be higher in A549 control tumor lysates. Treatment with BBR-SD formulation resulted in significantly down regulation of cyclin D1. The relative expressions were found to be reduced 1.02 and 8.10 fold significantly in BBR free drug and BBR-SD formulations, respectively compared to untreated control tumor lysates (Figure 6A&7A). The tumor suppressor protein p53 levels also indicated the promising anticancer effects of BBR in both free drug and SD formulations. Compared to untreated control, the relative expression of p53 was increased (2.51 and 3.76 fold) in BBR free drug and BBR-SD groups, respectively, BBR-SD produced 1.49 fold increased p53 expression compared to BBR free drug (Figure 6A&7B). The MMP-9 expression was significantly decreased in SD formulation compared to untreated control and BBR free drug treated groups, with 2.16 and 3.50 fold reduction respectively (Figure 6A&7C). The hypoxia inducible factor HIF-1α was also significantly down regulated in both BBR free drug and BBR-SD formulation treated tumors (Figure 6A&7D). All these results revealed that BBR in free drug form and in SD from significantly produced anticancer effects. In all parameters, except HIF-1α, BBR-SD formulation showed better anticancer effects than BBR free drug treated groups, which suggest the superior anticancer potential of our SD formulation.

### Immunohistochemical (IHC) analysis

**Figure 8:**
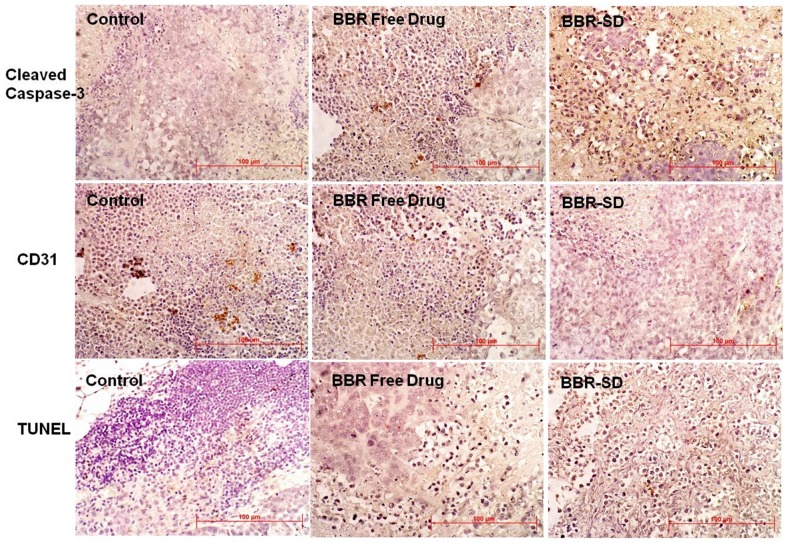
IHC analysis and VEGF levels. A) Quantification of cleaved caspase 3, CD31 positive MVD and TUNEL positive cells by IHC analysis. For IHC quantification, 10 fields were randomly counted from each tumor sections and 4 slides per group were used. B) VEGF levels in serum and tumor lysates after treatment with BBR free drug and BBR-SD. Each data point was represented as mean±sem (n = 3–4). *p<0.05 and ***p<0.001 Vs respective untreated control groups.

Further, the IHC analysis of tumor sections also supported the superior anticancer effects of BBR-SD formulation in A549 lung cancer model. The cleaved caspase 3 expressions was found to be significantly increased in BBR-SD formulations compared to untreated control and free drug treated groups (Figure 8).

**Figure 9:**
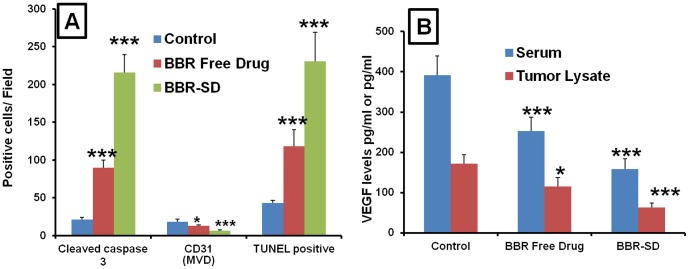
Histopathological Analysis. A) H&E stained histopathology of tumor section from orthotopic lung tumor models. B) H&E stained histopathological ileum sections after treatment with BBR free drug and BBR-SD. No visible toxicity was observed in GIT (Ileum). C) Body weights changes during the treatment of BBR free drug and BBR-SD, no significant change in the body weights were observed. Each data point was represented as mean±sem (n = 6–8).

**Figure 10:**
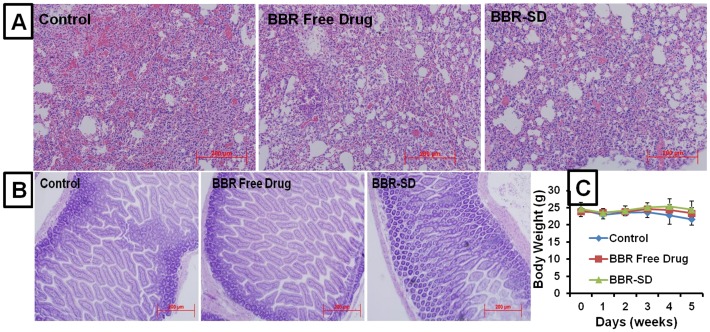
Anticancer effects of BA-SD formulations in orthotopic and metastatic lung cancer models. A) Lung tumor weights and tumor volumes after treatment with BA free drug and BA-SD formulations in A549 orthotopic and metastatic lung tumor models, B) Number of lung tumor nodules in peripheral, medial and central lobes in A549 metastatic models after treatment with BA free drug and BA-SD formulations. C) Quantification of TUNEL positive cells in orthotopic lung tumors D) Representative TUNEL assay images of orthotopic lung tumor images from control, BA free drug and BA-SD treated groups. Each data point was represented as mean±sem (n = 6–8). **p<0.01 and ***p<0.001 Vs respective untreated control groups.

Compared to control, the number of cleaved caspase 3 positive apoptotic cells was found to be 10.10 fold higher in BBR-SD formulation, whereas in BBR free drug group the number is 4.20 fold higher (Figure 9A). BBR-SD treatment resulted in 2.40 fold increased expression compared to BBR free drug, which suggest the superior anticancer effects of BBR-SD. Similarly, the CD31 positive cell expression was also significantly down regulated in BBR free drug and SD groups (Figure 8). The microvessel density (MVD) was found to be 1.44 and 2.75 fold reduced in BBR free drug and BBR-SD formulation groups compared to untreated control tumors (Figure 9A). The CD31 based MVD quantification also supported the observed superior anticancer effects of BBR-SD formulation. The significant increase in the TUNEL positive cell expression was observed in BBR-SD formulations compared to BBR free drug and control groups. Compared to control tumors, 2.74 and 5.36 fold increase in the TUNEL positive apoptotic cell expression was noticed in BBR free drug and BBR-SD formulations, respectively (Figure 8&9A), which suggest the role of apoptosis in BBR induced anticancer activity. Further, apoptosis was 1.95 fold increased in BBR-SD compared to BBR free drug group (Figure 9A). Histopathological H&E stain analysis of tumor sections also indicated the promising anticancer effects of BBR in free drug as well as in SD form (Figure 10A).

### VEGF estimation

The VEGF levels estimated by ELISA method suggested that VEGF levels were higher (597.90±54.03 pg/ml and 284.71±42.84 pg/mg of protein, respectively) in untreated control serum and in tumor lysates. Treatment with BBR free drug and BBR-SD formulation resulted in significant reduction (315.41±44.06 and 193.30±31.58 pg/ml, respectively) in the VEGF levels in serum. In tumor lysates, the VEGF levels were found to be 137.59±20.84 and 81.98±7.32 pg/mg protein respectively in BBR free drug and SD formulation treated groups (Figure 9B).

### TUNEL assay

The TUNEL assay also demonstrated that BA in free drug and BA-SD formulation significantly increased TUNEL positive apoptotic cells, which is responsible for the anticancer activity. TUNEL positive cells were found to be significantly higher in BA free drug and BA-SD treated animals compared to untreated control tumors. The quantitative analysis of TUNEL assay suggested that when compared to untreated control tumors, in BA free drug treated tumors 8.14 fold, whereas in BA-SD groups 15.14 fold increase in the apoptotic cells were observed. Further, BA-SD treatment resulted in 1.85 fold superior TUNEL positive apoptotic cells compared to BA free drug treated groups (Figure 5C&D).

### Real time PCR analysis

**Figure 11:**
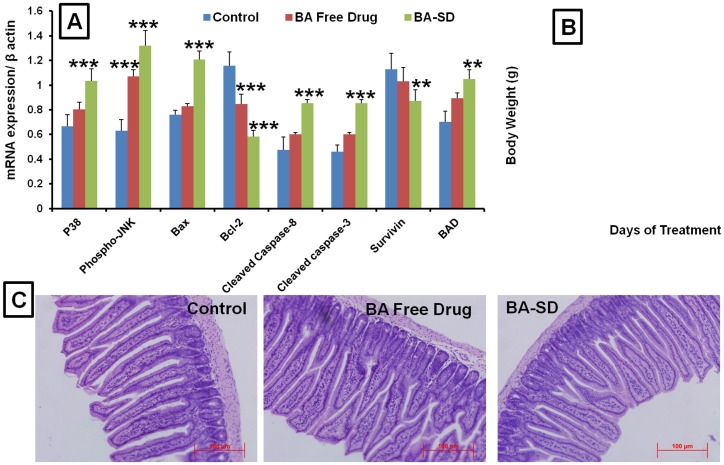
Real time-PCR analysis and Toxicity evaluation. A) Expression of p38, Phospho-JNK, Bax, Bcl-2, cleaved casapase-3&8, survivin and BAD in orthotopic lung tumor after treatment with BA free drug and BA-SD by real time PCR. B) Body weight changes during the treatment with BA free drug and BA-SD. C)Representative H&E stain based histopathological analysis of small intestine (jejunum) after treatment with BA free drug and BA-SD formulations. Each data point was represented as mean±sem (n = 6–8). **p<0.01 and ***p<0.001 Vs respective untreated control groups.

Real time PCR analysis was carried out to study the mechanism of anticancer effects of BA in free drug and BA-SD form. Compared to control groups, the expression of p38 (1.20 and1.55 fold), Phospho-JNK (1.69 and 2.09 fold) and Bax (1.08 and 1.58 fold) was significantly increased in BA free drug and BA-SD treated groups, respectively (Figure 11A). Antiapoptotic marker Bcl-2 expression was 1.36 and 1.98 fold significantly down regulated in BA free drug and BA-SD formulation treated groups compared to untreated control tumors. The proapoptotic markers cleaved caspase-3 and cleaved caspase-8 mRNA expressions were significantly increased in BA-SD treated animals compared to control tumors. Cleaved caspase-3 expressions were found to be 1.30 and 1.85 fold higher in BA free drug and BA-SD treated groups, respectively. The cleaved caspase-8 expressions were found to be 1.25 and 1.78 fold higher in BA free drug and BA-SD groups. The BAD expression was also 1.26 and 1.49 fold higher in free drug and SD formulations treated groups, compared to untreated control tumors. Further the cell survival marker survivin mRNA expression was 1.29 fold higher in BA-SD formulation group compared to untreated control tumors (Figure 11A). Overall, observations from mRNA expression studies, BA-SD produced better anticancer effects compared to same dose of free drug treated groups, which further confirm the superior anticancer effects of BA SD formulation.

### Effect of chronic administration of BBR and BA on toxicity

The chronic administration of BBR-SD and BA-SD formulation did not induce any signs of toxicity, because the body weight changes were found to be not altered among all three groups (Figure 10C & 11B). The H&E staining of small intestine ileum segments indicated no signs of toxicity on intestinal tract upon chronic administration of SD formulations containing various formulation excipients, indicating the safety of our SD formulation (Figure 10B & 11C). Similarly, the H&E staining of liver, lung, heart, spleen and kidney also resulted in no signs of toxicity (data not shown), suggesting that increased oral absorption of BBR in SD form did not induce any toxicity in vital organs.

## Discussion

In the current study, attempts have been made for the first time successfully to solve the poor oral bioavailabity problems associated with BBR and BA by using our novel dual channel spray drying formulation approaches [32]. We have intentionally chosen two natural products with different physicochemical properties as model drugs in an effort to discern the use of our dual channel spray gun technology in improving the pharmacokinetic and pharmacodynamic effects in vitro and in vivo. Due to its multidrug (P-glycoprotein) efflux pump and poor aqueous solubility, BBR has less than 1% low oral bioavailability. Because of its very poor bioavailability, the dose required for the therapeutic benefit is high [7], [10], [18]. Further, because of its promising biological activities, various strategies have been tried to improve the oral absorption of BBR [27], [33], [34]. On the other hand, most of the preclinical studies demonstrating the various biological effects of BBR have been reported with non-oral delivery approaches or used very high oral doses [35], [36]. Development of an oral formulation with improved oral pharmacokinetic properties may lead to significant therapeutic benefits. Discovery of BA as a growth inhibitor of melanoma cell lines and confirmation of its anticancer activity in various tumor models led to considerable interest in BA as an anticancer drug candidate [37]. BA is an inexpensive and available abundantly from common natural sources, notably the bark of white birch trees [38]. BA was found to be selective in killing transformed cancer cells, without affecting the normal cells [39]. The large difference between the doses required for BA anticancer activity and toxic side effects in animal models has generated interest in clinical development of this compound for cancer chemotherapy [40]. The selective targeting of cancer cells by BA provides the therapeutic window, which makes BA an attractive anticancer agent [39]. However, its biological effect is limited by its poor water solubility [29]. The oral doses used in anticancer studies in various preclinical animal models were higher (250–500 mg/kg). The limited aqueous solubility of BA leads to poor oral bioavailability which translates to its compromised therapeutic efficacy upon oral administration. Few formulation strategies have been attempted but none of them have been successful [17], .

In the present study, the BBR-SD formulations exhibited amorphous characteristics. After spray drying of BBR and BA formulation liquid mixture, particle sizes were in the micrometer ranges (1–6 µm). The significant increase (7.92 fold) in the release was observed in BA-SD formulation, suggesting that upon spray drying with the selected formulation components, the rate limiting step for poor oral absorption can be overcome. Further the mixing of the components through two streams from the dual channel spray gun also assists in a superior pharmaceutical product. In both BBR-SD and BA-SD formulation due to use of various formulation excipients, the in vitro drug release profile was found to be slow and sustained. In BA formulations, HPβ-CD and vitamin ETPGS incorporation increased solubility of BA. Carbopol acts as a control releasing agent and as bioavailability enhancer by prolonging the residence time of drug containing microparticles in GIT by its bioadhesive property. Further, the in vitro Caco-2 permeability studies revealed that the SD formulation of BBR resulted in significant increase in the apical to basolateral permeation of BBR. Permeation enhancers (Glycerol and menthone), and indirect permeation enhancer (chitosan) might have played role in improving the permeability of BBR. Further, use of vitamin E-TPGS and β- cyclodextrin through p-gp efflux pump inhibition or modulation might have also assisted in the increased permeability of BBR in invitro conditions. Hence a mixture of pharmaceutical excipients might increase the permeation of poorly permeable drugs better than the single excipient based formulation. In a recent study, 1,3-benzodioxole moiety of BBR when incorporated into β-cyclodextrin into an inclusion complex exhibited higher dissolution rate. Further, BBR-β-cyclodextrin complex resulted in increased intestinal absorption by Pgp modulatory activity [42]. Our studies are in agreement with the earlier studies demonstrating the role of Hydroxypropyl β-cyclodextrin on P-gp modulation to improve the oral absorption of BBR. In addition, use of glycerol as a permeation enhancer was also reported in previous studies [43]. As reported in several studies, the oral bioavailability of BBR in the free drug form was very limited [44]. The C_max_ and AUC _total_ values were found to be very low in the free drug groups. This might be the one of the reason why several clinical studies based on the oral administration of BBR failed to demonstrate the therapeutic benefits. The SD formulation of BBR resulted in significant increase in the oral absorption of BBR, when compared to free drug, the SD formulation resulted in the 3.46 fold and 6.98 fold increases in the C_max_ and AUC values, respectively.

Our results clearly demonstrate the effect of chitosan on increased oral absorption of BBR. Further, chitosan due to its gastro retentive or mucoadhesive ability might have increased the residence time of drug and the BBR-SD microparticles which might control the release of the drug for prolonged time in the GIT. The contribution of chitosan in increased absorption of BBR from chitosan containing formulation also is supported by a recent study, which demonstrated the role of mucoadhesive chitosan polymer on increased oral absorption of BBR [45]. The absorption enhancing ability of chitosan may be due to its ability to improve the BBR paracellular pathway in the intestinal tract. Similarly, incorporation of HPβ-CD, vitamin ETPGS, volpo-20 and carbopol resulted in significant increase in the BA dissolution profile. The increased BA dissolution also showed increased permeability in Caco-2 experiments. Further, the pharmacokinetic analysis of BA-SD formulations demonstrated significant increase in plasma AUC, C_max_, t_1/2_, MRT parameters compared to BA free drug. Plasma AUC levels were increased by 7.41 fold in BA-SD groups, which suggest the role of SD mediated improved BA solubility in increasing the bioavailability of BA-SD oral formulations. Similarly, previous studies have also demonstrated that bioavailability of BA can be increased by enhancing the dissolution rate and the solubility [41]. Since, spray drying mediated solid dispersions are mainly used to improve the aqueous solubility of poorly soluble drugs, our SD approach to improve the oral bioavailabity of BA is logical, which is also demonstrated with significant improvement in the oral pharmacokinetic profile. Our uniquely designed dual channel spray drying system allows the formation of drug containing microparticles, which are coated with mucoadhesive Chitosan (in BBR-SD) or carbopol (in BA-SD) polymers to increase the GIT residence time of the formulations. Overall, the increased solubility, sustained drug release from SD microparticles and mucoadhesive properties resulted in the significant increase in the oral bioavailability of BBR and BA. The anticancer effects of BBR-SD and BA-SD formulations also further support our pharmacokinetic observations. Based on the increased oral absorption, BBR-SD formulations were evaluated for anticancer activity in orthotopic lung tumor models. Upon oral administration of SD BBR formulation (100 mg/kg/day for 3 weeks), significant reduction in the lung tumor weights and volumes was seen. Our western blot results indicated that BBR showed anticancer activity through apoptosis and further increased p53 levels in BBR treated animals suggesting its role. BBR treatment resulted in significant reduction in the metastatic protein MMP-9. Our results are in agreement with the reported studies indicating the role of p53 in BBR mediated anticancer effects in colon cancer cells [46]. The role of HIF-1α in BBR induced anticancer effects was also observed in our studies. The IHC analysis of the tumors for micro vessel density (MVD) suggested that CD31 expression was significantly reduced in SD formulation treated groups compared to free drug treated groups, suggesting the antiangiogenic effect of BBR when the drug concentrations increased in the SD formulations. Both MVD and VEGF results indicate that BBR produced anticancer effects by inhibiting angiogenesis. Recently, similar kinds of studies were reported, in which the inhibitory effects of BBR on VEGF and HIF-1α expression were investigated to produce anticancer effects [11], [47]. The antimetastatic effect of BBR is evidenced from reduced expressions of MMP-9, which is in agreement with previous studies demonstrating the antimetastatic effects of BBR in breast cancer models [48].

The significant improvement of oral bioavailability of BA in BA-SD formulations translated into increased anticancer effects in orthotopic and metastatic lung tumor models. The lung tumor weights and volumes were significantly decreased in BA-SD formulations upon oral administration at the dose of 100 mg/kg, daily for 3 weeks. Interestingly, BA also found to be effective in metastatic lung tumor models, which makes BA more interesting drug candidate. In metastatic models, the numbers of tumor nodules in the peripheral, medial and central lobes were significantly decreased in BA treated groups. TUNEL assay indicated that BA produces anticancer activity through apoptosis, which was well documented in other studies as well [49]. Due to increased oral bioavailability, when compared to BA (free drug), BA-SD formulations produced superior anticancer cancer effects in terms of tumor weights, volumes and molecular studies also confirmed the pharmacodynamic effects of BA-SD. The RT-PCR studies further confirmed the superior anticancer effects of BA-SD formulation. Upregulation of p38 in BA-SD formulation suggested the role of p38 in BA induced apoptosis. Phospho-JNK expressions were also significantly increased in BA treated tumors. Our observations are further supported by other reports indicating the role of p38 and Phospho-JNK in BA induced apoptosis [49]. The proapoptotic markers Bax, cleaved caspase-8, cleaved caspase-3 and BAD were significantly increased in BA treated tumors. Further, the antiapoptotic markers Bcl-2 mRNA expression was reduced in BA-SD formulations suggesting the role of apoptosis in BA induced anticancer effects.

Upon administration of BBR and BA in free drug and SD form for 3 weeks at 100 mg/kg dose did not result in any toxicity which was confirmed from the body weights changes and histopathology. The H&E staining of GIT organs did not indicate any signs of toxicity of the long term dosing of free drugs or SD formulation ingredients suggesting the safety of the various ingredients used for the SD formulations. Since toxicity of these compounds is not evidenced, it is assumed these compounds are safer than the existing chemotherapeutic agents. Similar kinds of safety profiles have been demonstrated for these compounds in various studies [50], [51].

## Conclusions

Considering the challenging task of drug discovery and development, and apart from the pharmacological effects which represent the first development step, it is of great relevance to consider the biopharmaceutical and pharmacokinetics properties of the drugs of natural origin for further development. The limitation of poor permeability of BBR was successfully overcome by including the permeation enhancers like glycerol and menthone in SD formulations. The use of vitamin E TPGS and chitosan in the SD formulations resulted in significant increase in the oral bioavailability of BBR. Development of BA-SD formulation resulted in significant increase in the aqueous solubility and improved oral bioavailability. The increased oral absorption of these two drugs resulted in significant increase in anticancer effects in orthotopic and metastatic lung cancer models. Our results clearly demonstrate that SD formulation prepared using our patented technology of a dual channel spray gun is a superior alternative than other approaches for anticancer compounds like BBR and BA. Our SD formulation approaches opens new avenues delivery strategies to improve the therapeutic performance of orally active safe anticancer drugs.

## Supporting Information

Figure S1 **In vitro cytotoxicity of BBR.** The percentage viabilities of A549 and H1650 cell after treatment with different concentrations of BBR for 72 h. Each data point was represented as mean±sem (n = 6–8). *p<0.05, **p<0.01 and ***p<0.001 Vs respective untreated control groups.
(TIF)

Figure S2 **Effect of BBR on colony formation.** A) Effects of BBR free drug and BBR-SD on spheroid number B) on spheroid sizes. Representative clonogenic images of H1650 colonies form C) control and D) BBR free drug and BBR-SD treated groups. Colonies were stained with crystal violet staining for better visibility. Each data point is represented as mean±sem (n = 6–10). **p<0.01 and ***p<0.001 Vs respective untreated control groups.
(TIF)

Data S1 **Detailed experimental method description.** A) In vitro permeability studies, B) Western blot analysis, and C) IHC for cleaved caspase 3 and CD31.
(DOCX)
