# Supplementary figures and images for: Approaches to Improve the Oral Bioavailability and Effects of Novel Anticancer Drugs Berberine and Betulinic Acid
Source: PLoS One. 2014 Mar 10;9(3):e89919. doi: 10.1371/journal.pone.0089919 (PMC3948684; doi:10.1371/journal.pone.0089919)

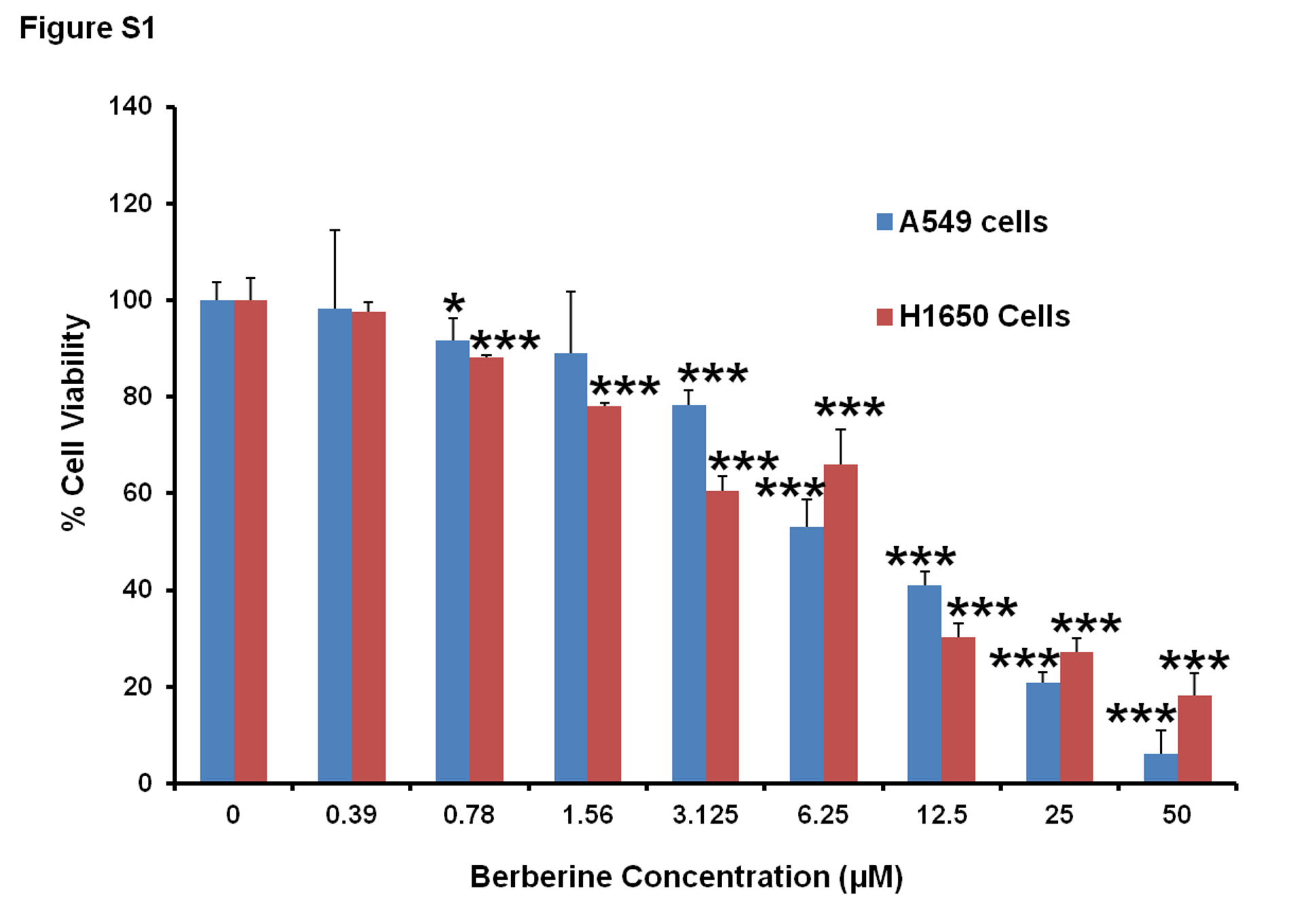

Supplement: Figure S1 — In vitro cytotoxicity of BBR. The percentage viabilities of A549 and H1650 cell after treatment with different concentrations of BBR for 72 h. Each data point was represented as mean±sem (n = 6–8). *p<0.05, **p<0.01 and ***p<0.001 Vs respective untreated control groups. (TIF) [file pone.0089919.s001.tif]

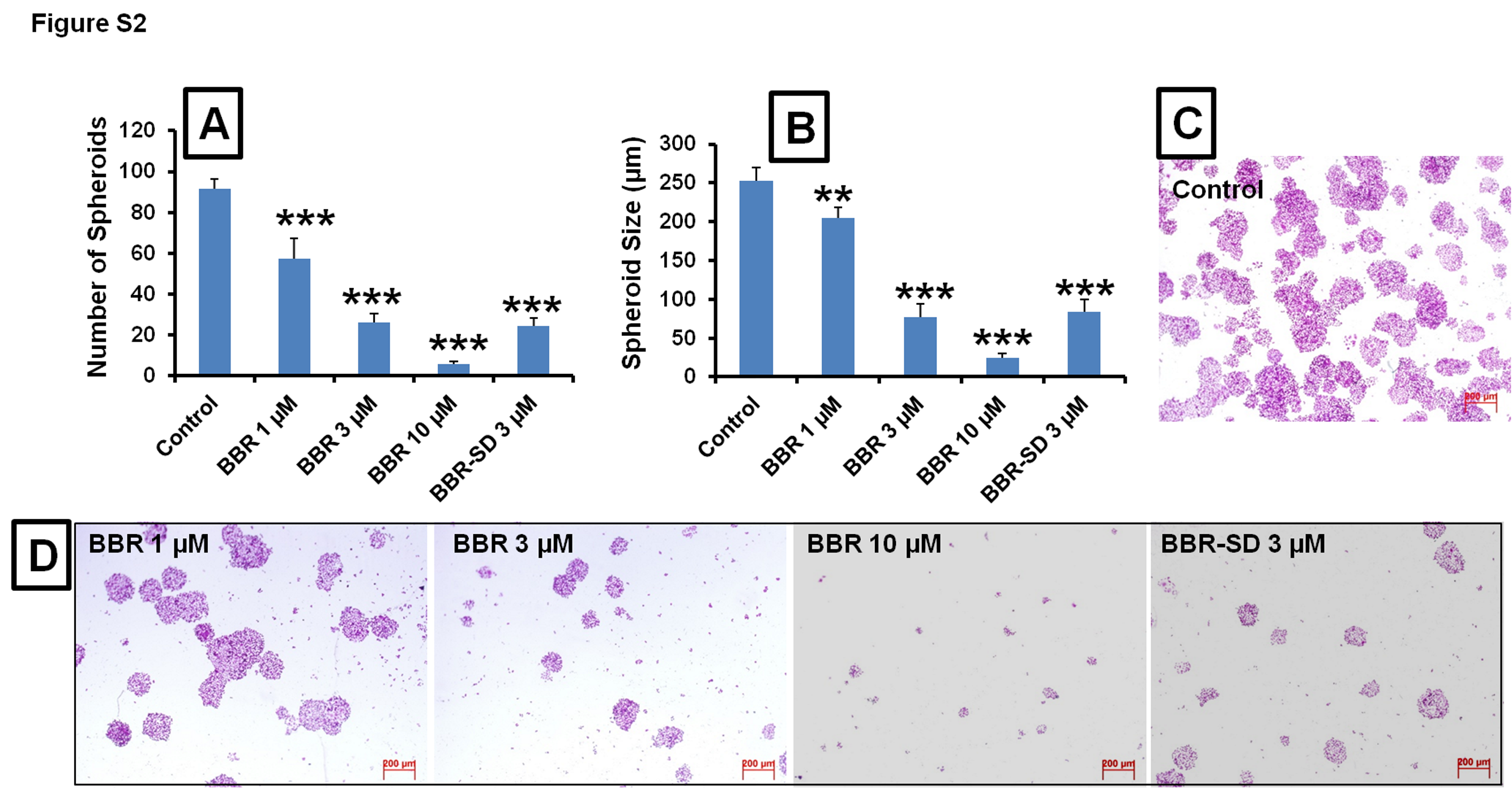

Supplement: Figure S2 — Effect of BBR on colony formation. A) Effects of BBR free drug and BBR-SD on spheroid number B) on spheroid sizes. Representative clonogenic images of H1650 colonies form C) control and D) BBR free drug and BBR-SD treated groups. Colonies were stained with crystal violet staining for better visibility. Each data point is represented as mean±sem (n = 6–10). **p<0.01 and ***p<0.001 Vs respective untreated control groups. (TIF) [file pone.0089919.s002.tif]
