# Supplementary material for: Approaches to Improve the Oral Bioavailability and Effects of Novel Anticancer Drugs Berberine and Betulinic Acid
Source: PLoS One. 2014 Mar 10;9(3):e89919. doi: 10.1371/journal.pone.0089919 (PMC3948684; doi:10.1371/journal.pone.0089919)
Supplement: Data S1 — Detailed experimental method description. A) In vitro permeability studies, B) Western blot analysis, and C) IHC for cleaved caspase 3 and CD31. (DOCX) [file pone.0089919.s003.docx]

**Data S1:**

***A) In vitro permeability studies:*** In vitro drug permeability studies were evaluated in Caco-2 cells according to the standard procedures, the detailed procedure was given supplementary material. detailed procedure. Caco-2 cells (passage #29) were cultured at 37°C with culture medium replaced every 2 days in an atmosphere of 5% CO2 and 95% relative humidity. Cells were harvested upon reaching approximately 80-90% confluence with 0.25% trypsin–EDTA. Cells were grown on 1.12 cm^2^ 0.4 μm pore polycarbonate membrane inserts in 12 mm × 12 transwell permeable support plates (Corning, NY, USA). Caco-2 cells were seeded onto membranes at 50,000 cells/well. Individual wells received 1.5 mL of culture medium at the bottom and 0.5 mL of medium was added to the top of inserts which was changed on alternate days. Confluent monolayers were used for permeability studies at ~21 days post seeding. Transepithelial electrical resistance (TEER) of Caco-2 monolayers was measured prior to all experiments using the EVOM volt-ohm-meter (Millicell-ERS; Millipore, USA) to ensure monolayer integrity. The TEER (Ω·cm^2^) values of the cell monolayers were determined by the resistance (Ω) × effective membrane area (cm^2^). TEER values were measured across each cell monolayer prior to beginning the experimentation and at the last sample collection time point. Transport studies were initiated by removing culture medium from the apical (A) and basolateral (B) sides of the cell monolayer. Cells were washed once with HBSS (37°C), and replaced with fresh 10 mM 2-(N-morpholino) ethanesulfonic acid (p*H* 5.0-5.8) or HEPES (p*H* 6.8-7.4) in HBSS, and equilibrated for 30 min. The volumes of A and B compartments were 0.5 and 1.5 mL, respectively. The monolayers were placed onto a plate shaker set at 30 rpm throughout the experiment to minimize the influence of the aqueous boundary layer. Cell monolayers were incubated for 120 min with continuous agitation at 37 °C with 100 µM of BBR and BA free drug solution and BBR-SD & BA-SD formulations (0.5 mL) in the donor compartment at p*H* 5.8. Samples were withdrawn from the receiver compartment after 120 min and drug concentrations were estimated by HPLC analysis. The paracellular integrity of monolayers was ensured based on low permeability of Lucifer yellow (a passive diffusional marker) and TEER consistently above 350 Ω·cm^2^. The effective permeability (*P*_eff_) (cm/s) was determined using Eq. 1:

| $P_{eff}=\frac{V_{R}}{A\times C_{o}}\times\frac{dC}{dt}$ |  |
| --- | --- |

where, *V*_R_ is the volume of the receptor chamber, *A* is the surface area of the filter, *C*_o_ is the initial donor concentration, and d*C*/dt is the slope of the cumulative concentration in the receiver chamber with time.

***B) Western blot analysis****:* western blot analysis was performed to study the expression of Bcl-2, cleaved caspase 3, survivin, cyclin D1, p53, MMP-9, HIF-1α. The detailed procedure was given in supplementary data. Protein was extracted from tumor tissues collected from untreated control and drug treated animals using RIPA lysis buffer (50mM Tris-HCL, pH 8.0, with 150 mM sodium chloride, 1.0% Igepal CA-630 (NP-40), 0.5% sodium deoxychlorate, and 0.1% sodium dodecyl sulfate) with protease inhibitor and 500 mM phenylmethylsulfonyl fluoride. Protein concentration was measured using BCA Protein Assay Reagent Kit (PIERCE, Rockford, IL). Equal amounts of supernatant protein (50 μg) was denatured by boiling for 5 min in SDS sample buffer, separated by 10% SDS-PAGE, transferred to nitrocellulose membranes for immunoblotting. Membranes were blocked with 5% skim milk in Tris-buffered saline with Tween 20 [10mM Tris-HCL (pH 7.6), 150 mM Nacl, and 0.5% Tween 20] and probed with antibodies against Bcl-2 (1:1000), cleaved caspase 3 (1:500), survivin (1:1000), cyclin D1(1:1000), p53 (1:1000), MMP-9 (1:1000), HIF-1α (1:1000) and β-actin (1:1000). Horseradish peroxidase-conjugated secondary antibodies (Santa Cruz Biotechnology, Santa Cruz, CA) were used. Proteins were visualized using enhanced chemiluminescent solution (Pierce, Rockford, IL) and exposed to Kodak X-OMAT AR autoradiography film (Eastman Kodak, Rochester, NY).

***C) IHC for cleaved caspase 3 and CD31:*** Formalin fixed tumors were processed for automatic histopathological procedures. The detailed procedure was provided in supplmmentary data (Supple). Tissue sections were dehydrated by immersing in increasing concentrations of alcohol and embedded in paraffin. Paraffin embedded specimens were cut into thin sections (4-5 µm). Tissue sections (4–5 μm thick) were mounted on poly-L-lysine–coated slides and sections were deparaffinized with xylene and rehydration through graded concentrations of alcohol, then incubated with 3% hydrogen peroxidase for 20 min to block endogenous peroxidase activity. Antigen retrieval for cleaved caspase 3 and CD31 staining was carried out for 10 min in 0.01 M sodium citrate buffer (pH 6), heated at 95°C in a steam bath followed by cooling for 30 min. Endogenous peroxidase was blocked by 3% hydrogen peroxide in PBS for 10 min. The slides were washed with PBS and incubated for 1h at room temperature with a protein blocking solution. Excess blocking solution was drained, and the samples were incubated overnight at 4°C with cleaved caspase 3 and CD31 antibodies or incubated with biotinylated secondary antibody followed by streptavidin. The color was developed by exposing the peroxidase to a substrate-chromagen, which forms a brown reaction product. The sections were then counterstained with hematoxylin. Cleaved caspase 3 and CD31 expression was identified by the brown cytoplasmic staining. Numbers of positive cells per filed was quantified by counting 10 different fields from each section. Based on the CD31 positive cells, microvessel density (MVD) was calculated ([30](#_ENREF_30)).
